# CuS-NiTe_2_ embedded phosphorus-doped graphene oxide catalyst for evaluating hydrogen evolution reaction

**DOI:** 10.1038/s41598-024-78870-w

**Published:** 2024-11-11

**Authors:** Sedigheh Parvaz, Zahra Talebi Vandishi, Ali A. Ensafi, Kimia Zarean Mousaabadi

**Affiliations:** 1 Department of Chemistry, Isfahan University of Technology, Isfahan, 84156-83111 Iran; 2 Department of Chemistry & Biochemistry, University of Arkansas, Fayetteville, AR 72701 USA

**Keywords:** Hydrogen evolution reaction, Bimetallic, Telluro-sulfide, Graphene oxide, Electrocatalyst, Electrochemistry, Energy

## Abstract

The hydrogen evolution reaction (HER), a crucial half-reaction in the water-splitting process, is hindered by slow kinetics, necessitating efficient electrocatalysts to lower overpotential and enhance energy conversion efficiency. Transition-metal electrode materials, renowned for their robustness and effectiveness, have risen to prominence as primary contenders in the field of energy conversion and storage research. In this investigation, we delve into the capabilities of transition metals when employed as catalysts for the HER. Furthermore, we turn our attention to carbon nanomaterials like graphene, which have exhibited tremendous potential as top-performing electrocatalysts. Nevertheless, advancements are indispensable to expand their utility and versatility. One such enhancement involves the integration of phosphorus-doped graphene. Our research focuses on the synthesis of CuS–NiTe_2_/PrGO, a nanocomposite with a crystalline structure, through a straightforward method. This nanocomposite exhibits enhanced catalytic activity for the HER, boasting a Tafel slope of 57 mV dec^−1^ in an acidic environment. Consequently, our findings present a straightforward and efficient approach to developing high-performance electrocatalysts for HER.

**Supplementary Information:**

The online version contains supplementary material available at 10.1038/s41598-024-78870-w.

## Introduction

Providing affordable, sustainable energy to meet the needs of a growing global population presents an immense challenge. Energy consumption worldwide is expected to increase substantially from current levels by 2040^1^. While fossil fuels like oil and coal still make up the majority of global energy use, concerns about their contribution to climate change have highlighted the need for renewable energy sources like solar and wind^2^. However, integrating these intermittent clean energy sources into our current electric grid remains a barrier^3^. An encouraging solution is using electrochemical water splitting (EWS) to store renewable energy as emission-free hydrogen fuel^4^. However, widespread implementation of water splitting will require substantial technological advancements to become economically viable. In particular, highly active and stable electrocatalysts made from abundant, low-cost materials need to be developed^5^.

Generally, EWS involves two half-reactions, HER and oxygen evolution reaction (OER), which need efficient electrocatalysts to reduce the overpotential and accelerate the reaction. Traditionally, platinum and the oxides of iridium and ruthenium are the best candidates for HERs and OERs^6^. In recent years, there has been a shift towards using abundant transition metal (TM)-based catalysts instead of scarce and expensive platinum group metals (PGMs) for the HER. This has led to the development of various TM-related electrocatalysts like carbides^7^, phosphides^8^, and chalcogenides^9^ that can match the HER performance of PGMs. Among these promising catalysts, TM tellurides (TMTs) have generated significant interest due to the reduced electronegativity, but higher metallic characteristics of Te compared with other chalcogenides like O, S, and Se^10^. They exhibit remarkable stability, numerous active sites, excellent conductivity, low overpotential, and strong electronic interactions between material components, all contributing to improved catalytic performance^10^.

TM sulfides (TMSs) have been extensively researched as alternative electrocatalysts to replace costly platinum for the HER. This is due to TMSs having similar electronic band structures as noble metals^11^. Some of the most studied TMSs as potential Pt replacements for HER include NiS_2_^12^, FeS_2_^13^, WS_2_^14^, and CuS^15^. Among these promising TMS candidates, CuS has shown particular advantages as an HER electrocatalyst owing to its electrocatalytic and photocatalytic properties^11^. CuNi bimetallic electrocatalysts are low-cost non-noble metals that have been previously used for hydrogen storage and HERs^16^. For instance, research on Chevrel-phase Mo_6_ × _8_ (X = Te, S, and Se) nanocatalysts for HER utilized the Gibbs free energy of hydrogen adsorption (ΔGH) as a descriptor for HER activity. Notably, it was found that as the electronegativity of the X-chalcogenide increases in Mo_6_ × _8_, the strength of hydrogen adsorption also increases^17^. Similarly, Lee et al. demonstrated that many TMDs, with appropriate anion-vacancy densities (such as MoTe_2_, TiTe_2_, and ZrTe_2_), rank high on the volcano plot, enhancing hydrogen bonding and promoting HER activity^18^. Moreover, studies have shown that CuNi composites exhibit improved HER performance compared to the individual Cu and Ni counterparts^19^. The enhancement in HER activity observed for CuNi bimetallic can be attributed to several factors including, preferential segregation of the most catalytically active metal to the surface, modifications in electronic properties, enhancement of larger electrochemically active surface area (ECSA) areas, or a combination of these effects^20^.

Besides, reduced graphene oxide (rGO) has emerged as a promising electrocatalyst material due to its high electrical conductivity, efficient charge transfer, and high specific surface area^21^. However, rGO sheets tend to agglomerate due to van der Waals forces, limiting their performance^22^. Doping rGO with heteroatoms such as phosphorus introduces more active sites and enhances electronic properties by increasing defect-induced active surfaces and local charge density^23^. Among heteroatoms, phosphorus is particularly effective as it has higher electronic modulation efficiency from its lone electron pairs^24^.

Embedding metals in GO also leads to outstanding electrocatalytic performance for reactions like the HER^25^. This synergistic effect arises from the large surface area, excellent conductivity, and tunable reactivity of graphene. Amorphous electrocatalysts often outperform crystalline counterparts, as their structural defects confer notable activity improvements^26^. Overall, PrGO and crystalline metal-embedded graphene hybrids demonstrate great promise as electrocatalysts due to their synergistic effects creating abundant active sites, superior conductivity, and diversified reactivity.

In this study, A novel CuS–NiTe_2_/PrGO was synthesized. First, PrGO was hydrothermally prepared. Then, copper, nickel, tellurium, and sulfur precursors were added and composited in situ on the PrGO substrate to obtain the CuS–NiTe_2_/PrGO nanocomposite material. The electrochemical performance of the synthesized CuS–NiTe_2_@PrGO composite was investigated for HER catalysis in acidic media. This structure offers abundant active sites, enhanced conductivity, and strong electronic interactions, which improve catalytic efficiency and stability. Additionally, this composition allows fine-tuning of hydrogen adsorption energy, leading to low overpotential and improved reaction kinetics. This composite incorporates the benefits of both sulfides and tellurides hence enhancing the HER performance. CuS improves on the electronic interactions as a result of its distinct electronic system whereas NiTe_2_ enhances stability and efficiency of the catalysts’ performance during HER^27,28^.

## Results and discussion

**Fig. 1:**
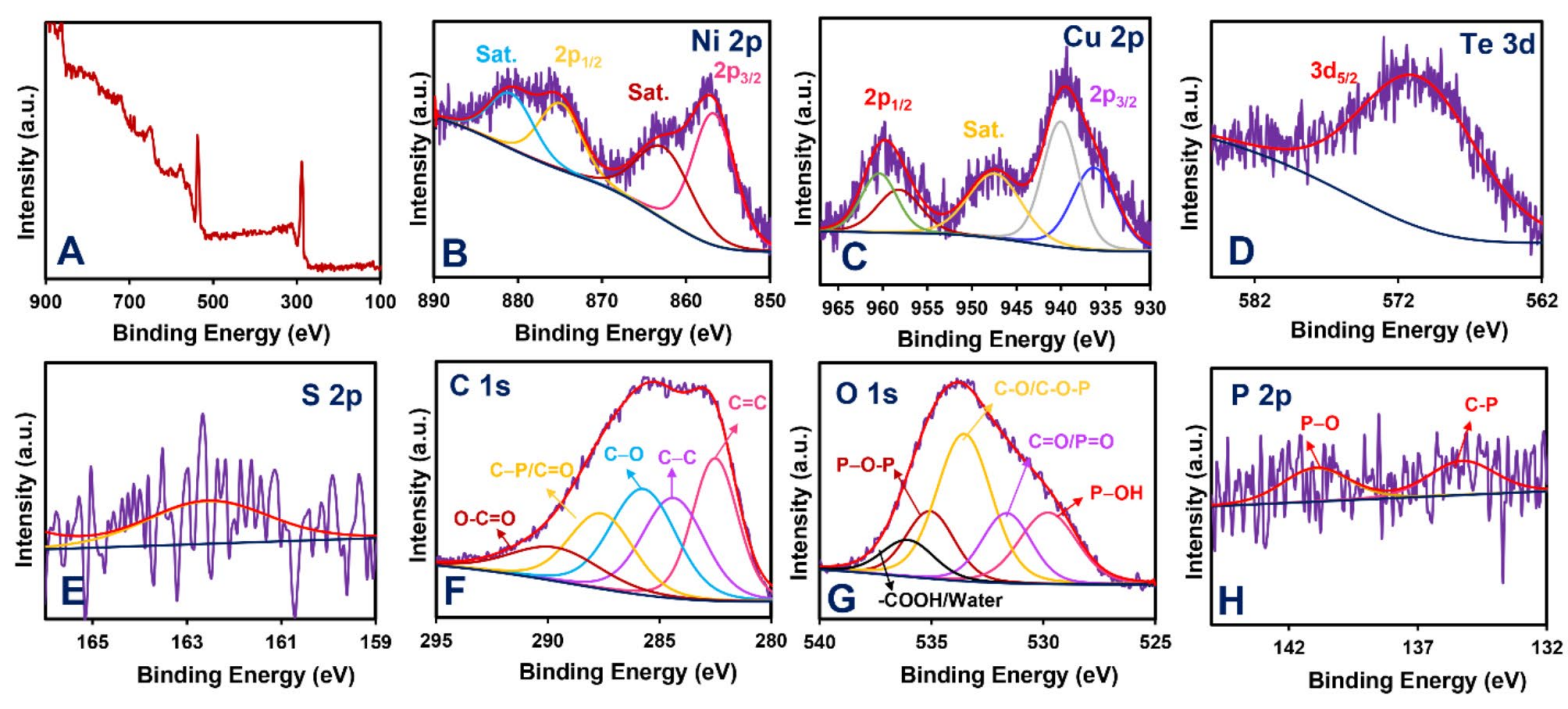
(**A**) XPS spectrum of CuS–NiTe_2_@PrGO. The high–resolution XPS spectra of (**B**) Ni 2p, (**C**) Cu 2p, (**D**)Te3d, (**E**) S 2p, (**F**) C 1s, (**G**) O 1s, and (**H**) P 2p.

The CuS–NiTe_2_@PrGO electrocatalyst’s XPS survey spectrum is shown in Fig. 1A. The spectra displayed the peaks of Cu, Ni, Te, S, O, P, and C. The high–resolution Ni 2p XPS spectrum of CuS–NiTe_2_@PrGO is shown in Fig. 1B. This spectrum exhibits two discernible peaks, positioned at 855.8 eV and 873.6 eV, corresponding to the Ni 2p_3/2_ and Ni 2p_1/2_ respectively, with a separation of 17.8 eV. This distinct peak separation unequivocally indicates the presence of nickel in its bivalent Ni^2+^ state^29,30^. The high–resolution Cu 2p XPS spectra of CuS–NiTe_2_@PrGO can be seen in Fig. 1C. The asymmetric Cu 2p peaks are due to the different chemical states of copper in CuS–NiTe_2_. The peak at 937.0 eV for Cu 2p_3/2_ and the peak at 958.0 eV for Cu 2p_1/2_, with a separation of 21.0 eV between them, are indicative of monovalent Cu^+^. The peaks at 940.5 eV for Cu 2p_3/2_ and 962.0 eV for Cu 2p_1/2_, with spin–orbit splitting, are attributed to bivalent Cu^2+^^31^. The occurrence of dual oxidation states of copper (Cu^+1^ and Cu^+2^) in CuS is supported by various existing studies. Specifically, in hexagonal CuS, the tetrahedral site contains Cu^+2^ centers, while the trigonal site contains Cu^+1^ centers. These Cu^+1^ centers are exclusively linked to monosulfides, whereas the Cu^+2^ centers at the tetrahedral sites are linked to both mono– and disulfides^32^. Moving to Fig. 1D, the high–resolution Te 3d XPS spectrum of CuS–NiTe_2_@PrGO is displayed, revealing a Te 3d peak at approximately 571.2 eV, signifying the presence of NiTe_2_^33^. Figure 1E presents the high–resolution S 2p XPS spectrum of CuS–NiTe_2_@PrGO where the peaks observed at 162.8 eV correspond to the S 2p peak attributed to S^[2–32^. In Fig. 1F, the high–resolution C 1 S XPS spectrum is presented. The peaks at 283, 284.4, 286.2, 278.9, and 290.5 can be attributed to C = C, C − C, C − O, C − P/C = O, and O − C = O. The O 1s peaks in Fig. 1G located at 530.1, 531.8, 533.8, 535.1, and 535.9 eV correspond to P − OH, C = O/P = O, C − O/C–O − P, P − O−P, and water/carboxylic groups, respectively^21^. Finally, the high–resolution P 2p XPS spectrum in Fig. 1H represented two peaks at 134.7 eV (C–P bond) and 139.3 eV (P–O bond)^21^. Therefore, the results confirm the successful incorporation of phosphorus into the graphene lattices. As stated in the introduction, the reversible redox reactions of P–O and C–P groups can increase overall energy density and thus improve electrochemical performance.

**Fig. 2:**
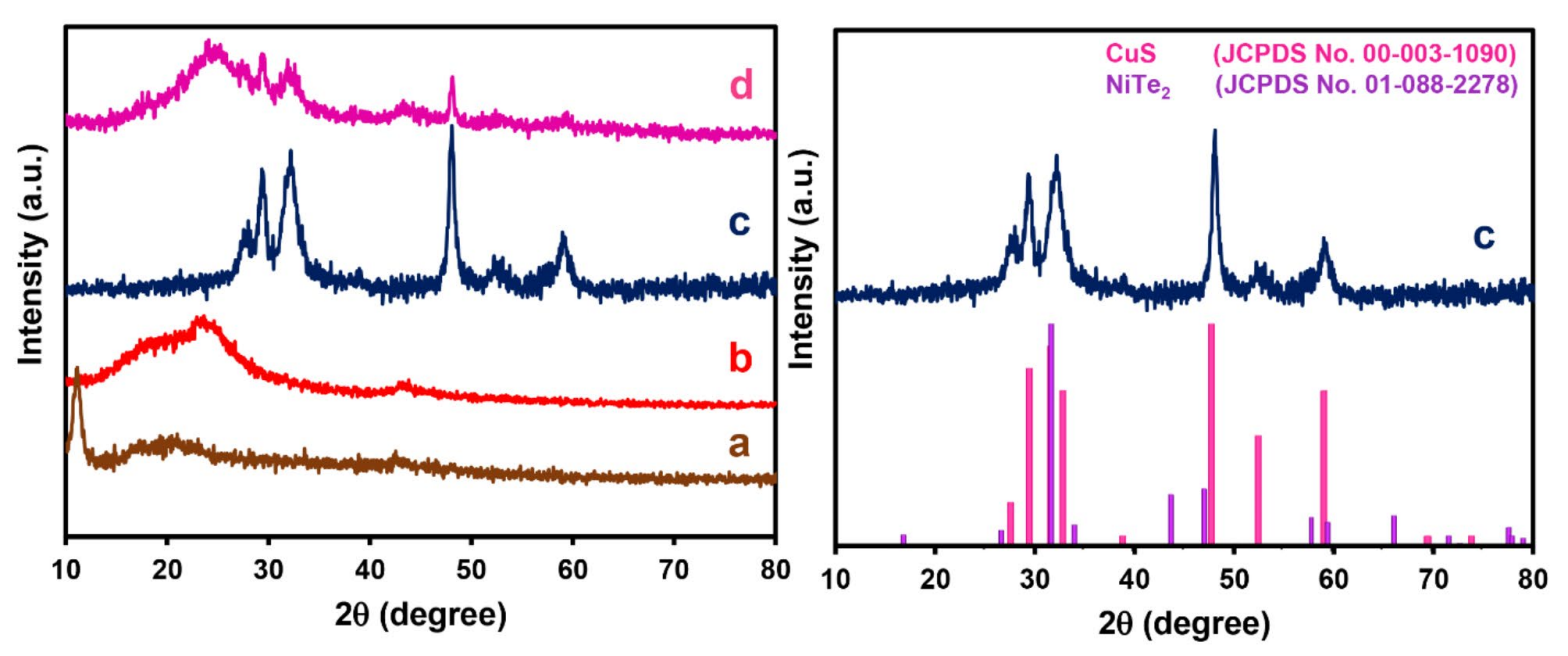
XRD patterns of (**a**) GO, (**b**) PrGO, (**c**) CuS–NiTe_2_, and (**d**) CuS–NiTe_2_@PrGO. (Patterns (a) and (b) reproduced from Ref.^34^)

The XRD patterns of GO and PrGO are compared in Fig. 2(left) (These patterns were used from our previous study^34^). The GO sample (spectrum a) displays a distinct diffraction peak at 2θ = 11.2°, indicative of the typical (001) crystalline planes of GO. In contrast, this peak is absent in the PrGO sample (spectrum b). Instead, two new broad peaks emerge at approximately 23.9°and 43.2° in the PrGO pattern, corresponding to the (002) and (101) planes, respectively. The emergence of these peaks suggests the reduction of GO has occurred, and the material now consists of a few stacked graphene layers rather than a single sheet. The broad, low–intensity peaks indicate poor stacking between the graphene sheets^35^. The XRD patterns of CuS–NiTe_2_ and the standard reference spectra for CuS (JCPDS No. 00–003–1090) and NiTe_2_ (JCPDS No. 01–088–2278) are shown in Fig. 2(right). The major diffraction peaks observed for CuS–NiTe_2_ at 2θ values of 16.2°, 27.62°, 28.17°, 29.51°, 32.32°, 35.9°, 48.08°, 52.45°, 59.16°, and 65.8° of CuS structure and hexagonal NiTe_2_ structure. The CuS–NiTe_2_ peak positions align well with the CuS and NiTe_2_ references, with only small shifts for the peaks at higher angles, possibly due to factors such as crystal lattice strain, particle size effects, and crystallographic orientation variations. In spectrum d both patterns of CuS–NiTe_2_ and PrGO were seen.

**Fig. 3:**
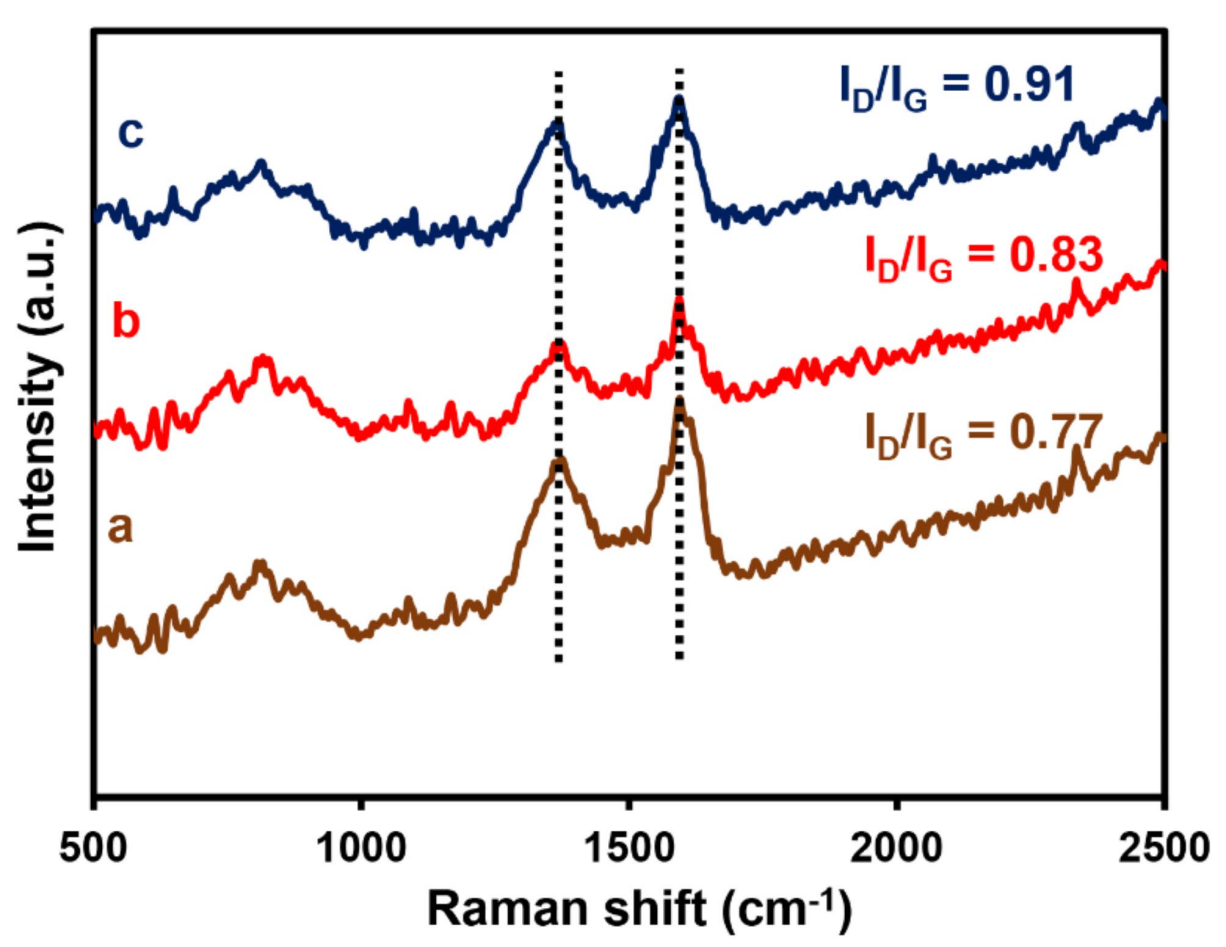
Raman spectra (**a**) GO, (**b**) PrGO, and (**c**) CuS–NiTe_2_@PrGO.

In order to further understand the specific chemical structures of the as–prepared composites, Raman spectroscopy was employed. As depicted in Fig. 3, two characteristic peaks are observed in the Raman spectra: the D band at approximately 1375 cm^–1^ and the G band at roughly 1594 cm^–1^. Compared to pristine GO, the G band exhibits a red shift in the spectrum for PrGO. This small red shift of the G band signifies altered electronic properties of the graphene upon phosphorus doping^36^. Additionally, the intensity ratio between the D and G bands (I_D_/I_G_) increases for both PrGO and the CuS–NiTe_2_@PrGO composite in contrast to pristine GO. The rise in I_D_/I_G_ indicates changes in the defects present in GO after phosphorus doping and in PrGO after composite with CuS–NiTe_2_ structure^36,37^.

**Fig. 4:**
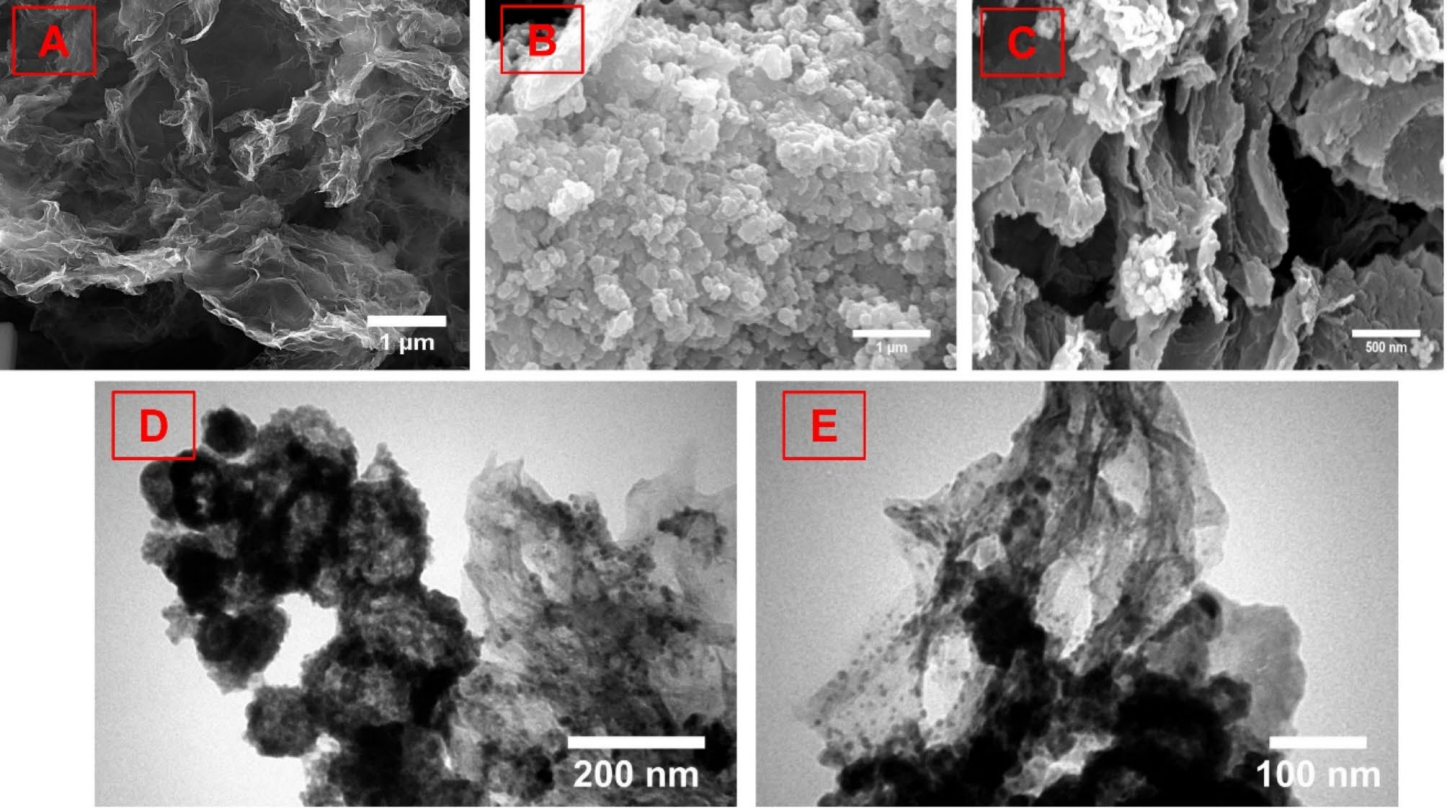
FE–SEM images of (**A**) PrGO (Reproduced with permission from Ref.^38^), (**B**) CuS–NiTe_2_, and (**C**) CuS–NiTe_2_@PrGO. TEM images of (**D**–**E**) CuS–NiTe_2_@PrGO.

The FE–SEM was utilized to investigate the morphological features of the synthesized electrocatalysts. As can be seen, the PrGO image exhibits a 3D porous network of GO sheets with an effective surface area making it a suitable substrate for growing and stabilizing CuS–NiTe_2_ composite (Fig. 4A) (This image was used from our previous study^38^). The structure of CuS–NiTe_2_ nanocomposite is displayed in Fig. 4B, it can be seen that highly dense and small nanoplates of CuS–NiTe_2_ are present. Figure 4C depicts the formation of the CuS–NiTe_2_@PrGO nanocomposite and the placement of CuS–NiTe_2_ nanoplate between the plates of PrGO. This porous architecture with 3D structure enhances HER kinetics by providing more active sites, shortening ion diffusion paths, and enabling facile charge transfer. TEM was conducted to further characterize the structural properties of CuS–NiTe_2_@PrGO. Figure 4D–E presents TEM images of CuS–NiTe_2_@PrGO. The TEM analysis displays that nanoplates of CuS–NiTe_2_ have formed on the transparent PrGO substrate, resulting in a three–dimensional nanostructure. Based on these findings, it can be inferred that the targeted CuS–NiTe_2_@PrGO nanocomposite was successfully synthesized via the synthesis method used.

**Fig. 5:**
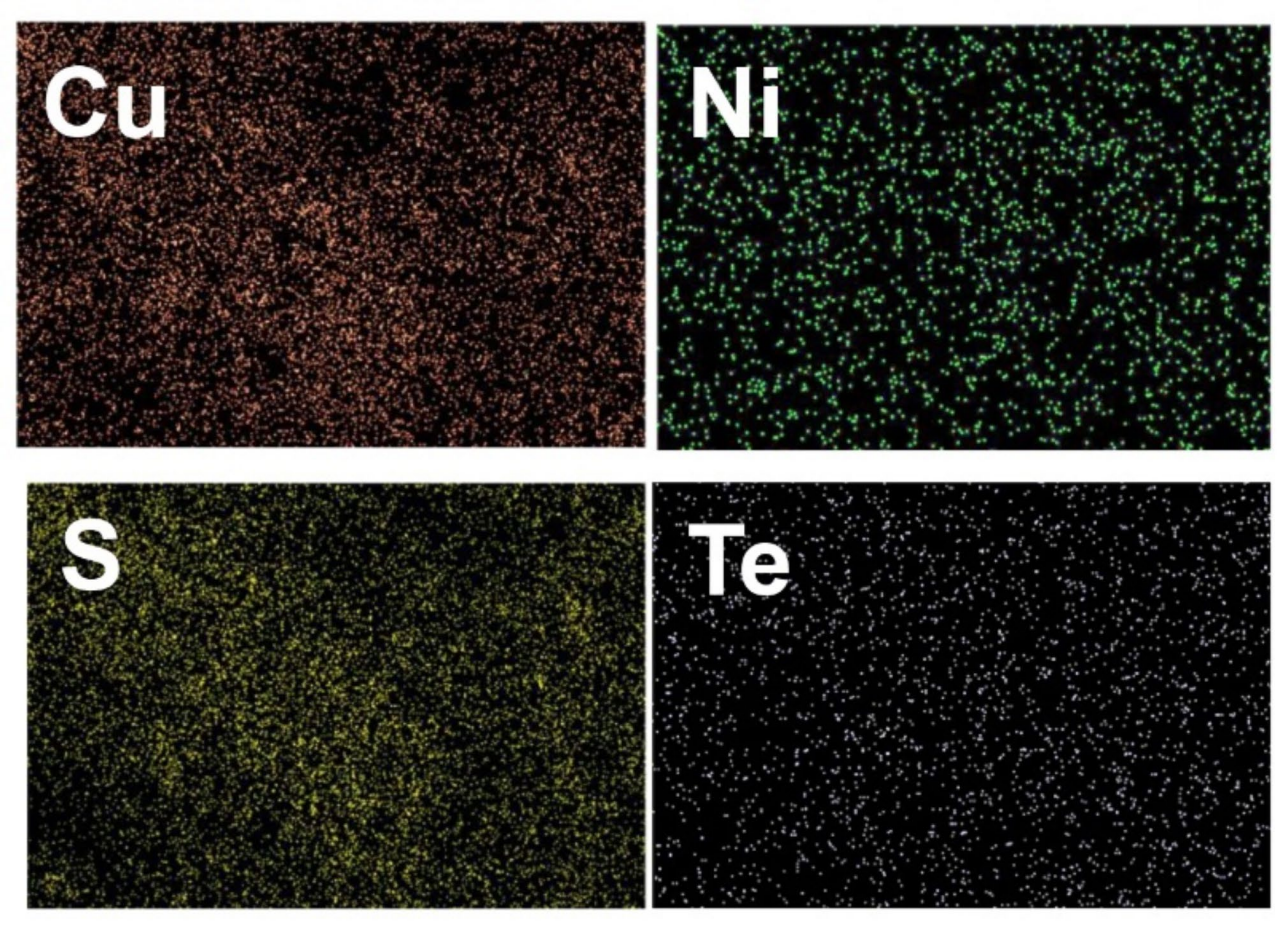
Elemental mapping of CuS–NiTe_2_.

Elemental mapping results depicted in Fig. 5 demonstrate that the synthesized CuS–NiTe_2_ structure contains Cu, Ni, Te, and S constituents with appropriate distribution.

**Fig. 6:**
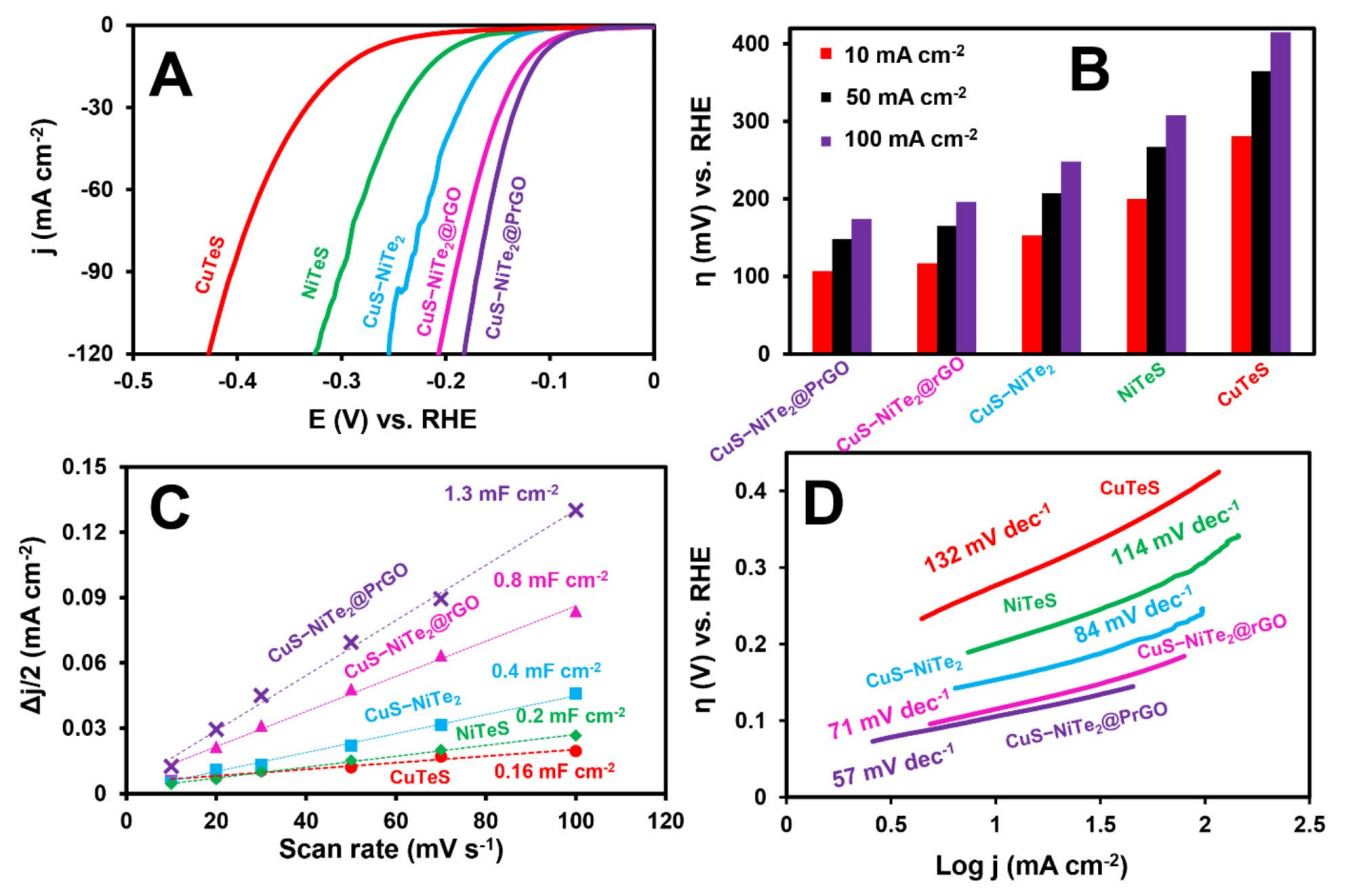
(**A**) Polarization curves at a scan rate of 10 mV s^–1^ in 0.5 M H_2_SO_4_, (**B**) overpotential at various current densities, (**C**) relationship of Δ*j*/2 vs. scan rates (**D**) semi–logarithmic cathodic current–potential curves of (a) CuS–NiTe_2_@PrGO, (b) CuS–NiTe_2_@rGO, (c) CuS–NiTe_2_, (d) NiTeS, and (e) CuTeS.

The CuS–NiTe_2_@PrGO nanocomposite’s effectiveness as an electrocatalyst for the HER was assessed through a LSV test conducted in a 0.5 M H_2_SO_4_ solution at room temperature. When evaluating HER electrocatalysts, two key parameters of interest are a lower overpotential at a specific current density and a lower Tafel slope. These parameters are indicative of the catalyst’s efficiency and performance. In Fig. 6A, it is evident that CuS–NiTe_2_@PrGO outperformed other tested samples by exhibiting the lowest overpotential at 10 mA cm^–2^, measured at 106 mV, in driving the HER.

Furthermore, for a comprehensive comparison between various samples, Fig. 6B illustrates the overpotential values of CuS–NiTe_2_@PrGO, CuS–NiTe_2_@rGO, CuS–NiTe_2_, NiTeS, and CuTeS at different current densities, including 10, 50, and 100 mA cm^–2^. This data helps in assessing how each sample performs under the same operating conditions and highlights the superiority of CuS–NiTe_2_@PrGO as an HER electrocatalyst due to its notably lower overpotential, signifying its efficiency in facilitating the HER process.

In other words, the synergistic effect between Cu and Ni in the CuS–NiTe_2_@PrGO nanocomposite plays a crucial role in enhancing its HER performance. Both Cu and Ni have favorable electronic structures that make them excellent catalysts for the HER. Specifically, Cu can adsorb and activate protons (H^+^ ions), while Ni facilitates the hydrogen adsorption and desorption steps. When combined in the nanocomposite, Cu and Ni can work cooperatively to accelerate the HER kinetics. This cooperative action reduces the energy barrier for the reaction, resulting in a lower overpotential, which is a key indicator of the electrocatalyst’s efficiency in promoting the HER.

PrGO showcases its prowess through a unique combination of attributes. With its enhanced catalytic activity stemming from the introduction of phosphorus atoms into the graphene lattice, PrGO lowers the overpotential and speeds up reaction kinetics. Its proficiency in facilitating electron transfer, coupled with the generous surface area for catalytic sites, ensures high efficiency in hydrogen production. Furthermore, the introduction of phosphorus into the GO structure led to an overall enhancement in charge density, exerting a positive influence. PrGO’s resilience against the rigors of the electrochemical environment and the flexibility to fine–tune its properties further solidify its position as a remarkable catalyst, promising to drive advancements in sustainable energy technologies with its remarkable catalytic potential.

The double–layer capacitance (C_dl_) was utilized to assess the ECSA of five different materials: CuS–NiTe_2_@PrGO, CuS–NiTe_2_@rGO, CuS–NiTe_2_, NiTeS, and CuTeS. To achieve this, cyclic voltammetry (CV) curves were recorded within the 0.1 to 0.3 V voltage range at various scan rates. As shown in Fig. 6C, the C_dl_ values for these materials are as follows: CuS–NiTe_2_@PrGO: 1.3 mF cm^−2^, CuS–NiTe_2_@rGO: 0.8 mF cm^−2^, CuS–NiTe_2_: 0.4 mF cm^−2^, NiTeS: 0.2 mF cm^−2^, and CuTeS: 0.16 mF cm^−2^, with CuS–NiTe_2_@PrGO exhibiting the highest C_dl_ value. The ECSA was determined by dividing C_dl_ by the specific surface capacitance (C_s_) using a commonly adopted Cs value of 0.035 mF cm^–2^, which is typically associated with metal surfaces^39,40^. CuS–NiTe_2_@PrGO demonstrates an elevated ECSA, owing to its abundant active sites, thereby augmenting its performance in the HER. Moreover, an analysis of the Tafel slope, derived from the semi–logarithmic cathodic current–potential curves, revealed that CuS–NiTe_2_@PrGO exhibited rapid kinetics in contrast to other modified electrodes (as illustrated in Fig. 6D).

The slope indicated that the HER mechanism followed the Volmer–Heyrovsky pathway, involving a series of electrochemical steps leading to the generation of gaseous hydrogen (H_2_) at the electrode surface (2 H^+^ +2 e^–^→H_2_). The initial step, the Volmer reaction (H_3_O^+^ + e^–^ + M ⇌ M–H_ads_ + H_2_O), is followed by the Heyrovsky reaction (M–H_ads_+ H_3_O^+^ + e–⇌ M + H_2_ + H_2_O), which culminates in the production of hydrogen gas. Here, “M” denotes the active site on the electrode surface. The rapid proton–electron transfers are facilitated by the proper binding of adsorbed hydrogen atoms (H_ads_) to CuS–NiTe_2_@PrGO, which readily dissociates to form H_2_ molecules, aligning with the principles of the Sabatier principle. Additionally, Table 1 provides a summary of key parameters, including the Tafel slope, cathode exchange current densities (j_0_), transfer coefficient (α), onset potential (E_on_), and overpotential (η), for the various modified electrodes.

**Table 1:** Tafel slope, exchange current densities at the cathode, transfer coefficient, onset potential, and overpotential were derived from the polarization curves of various modified electrodes.

Working electrode	Tafel slope (mV dec^–1^)	j_0_ (mA cm^–2^)	α	E_on_ (mV)	η_10_ (mV)
CuS–NiTe_2_@PrGO	57	0.14	1.04	–40	–106
CuS–NiTe_2_@rGO	71	0.24	0.84	–46	–116
CuS–NiTe_2_	84	0.17	0.70	–61	–152
NiTeS	114	0.21	0.52	–74	–199
CuTeS	132	0.081	0.45	–115	–280

**Fig. 7:**
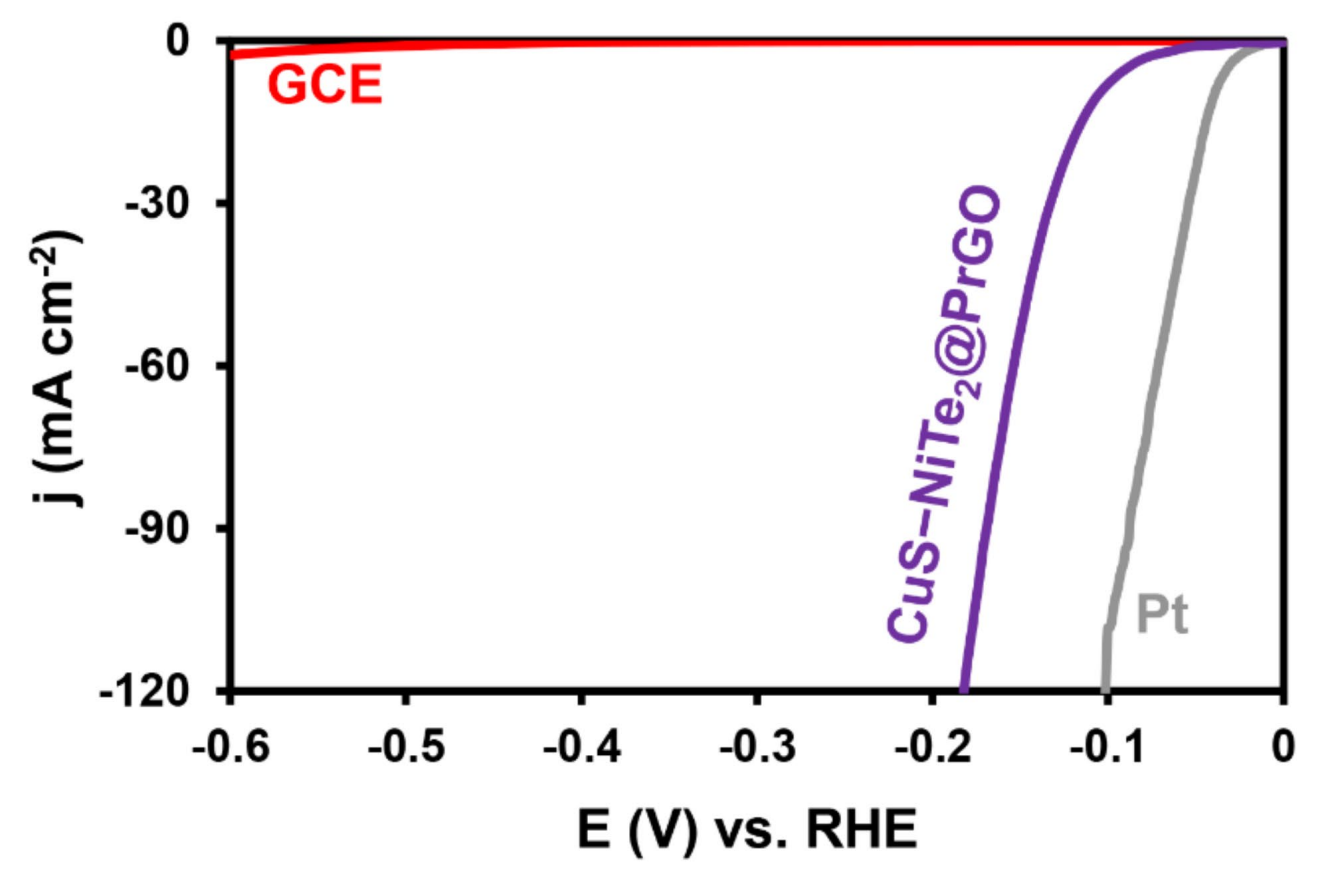
Polarization curves of Pt commercial, CuS–NiTe_2_@PrGO, and GCE.

Furthermore, the electrocatalytic performance of CuS–NiTe_2_@PrGO was assessed in comparison to a bare GCE and a commercial Platinum electrode under identical conditions. As depicted in Fig. 7, the GCE, when used as the working electrode, exhibited a negligible electrocatalytic performance. In contrast, CuS–NiTe_2_@PrGO demonstrated reasonably efficient performance in the HER when compared to the commercial Pt electrode.

EIS was employed to investigate the electron transfer kinetics of HER.

**Fig. 8:**
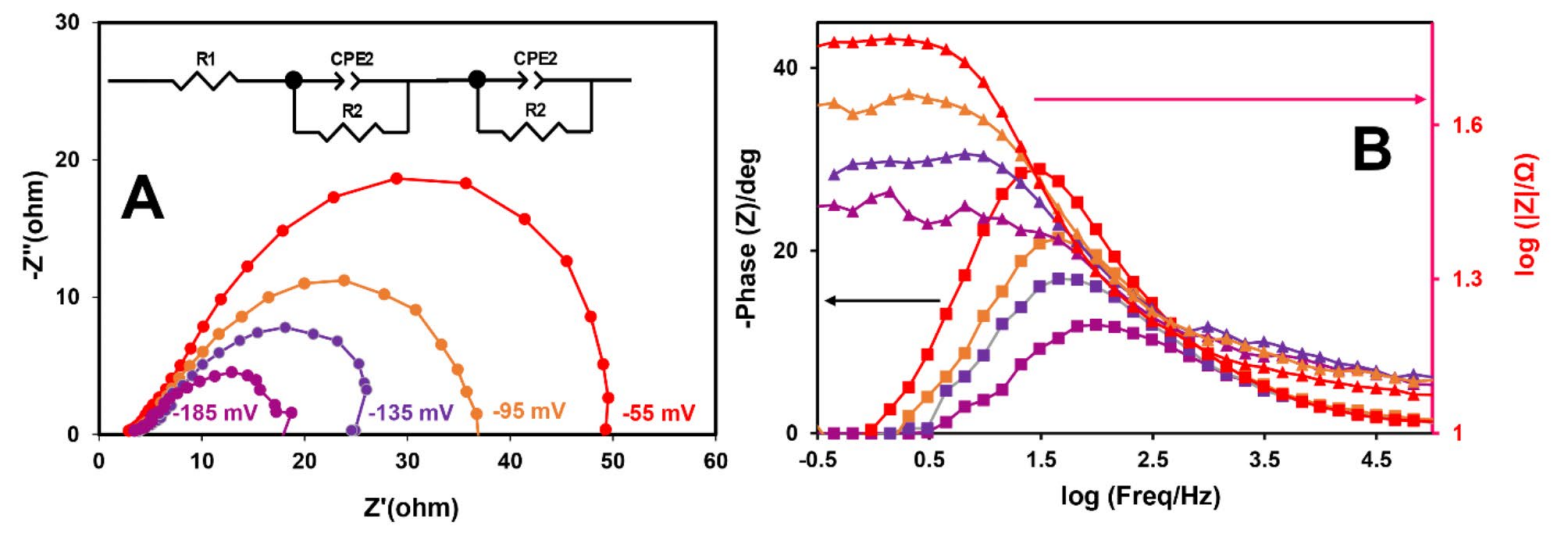
(**A**) Nyquist Plots of CuS–NiTe_2_@PrGO at the different applied potentials (inset: equivalent circuit), (**B**) Corresponding Bode plots.

Figure 8A displays Nyquist plots for CuS–NiTe_2_@PrGO at various applied potentials after 1000 sweeps. After 1000 sweeps, CuS–NiTe_2_@PrGO exhibited improved kinetics and charge transfer. Increasing the applied potential led to a reduction in the semicircle radius of the Nyquist curves, indicating that the HER process was primarily governed by charge transfer, consistent with prior findings^41^. Figure 8B presents corresponding Bode phase plots for all curves, revealing a phase angle at low frequencies associated with the HER kinetics^42^. As the applied potential increased, the phase angle decreased and shifted to higher frequencies, signifying faster reaction kinetics and reduced resistance. The results from polarization curves and EIS were in good agreement. The equivalent circuit for CuS–NiTe_2_@PrGO is shown in Fig. 8A (inset). Figure S1 shows the Nyquist plots of the CuS–NiTe_2_@PrGO, CuS–NiTe_2_@rGO, CuS–NiTe_2_, NiTeS, and CuTeS were acquired at − 135 mV vs. RHE. The minimum semicircle radius is attributed to CuS–NiTe_2_@PrGO, indicating the lowest charge transfer resistance (R_ct_) due to the fastest electron transfer rate.

**Fig. 9:**
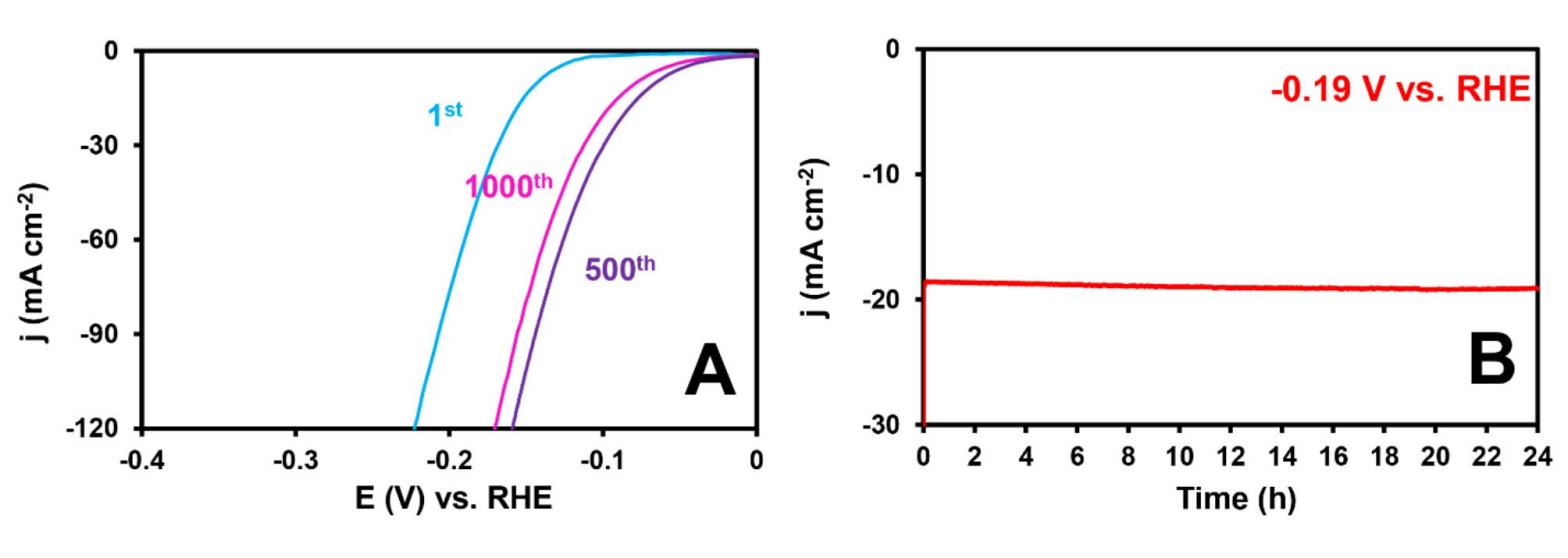
(**A**) Polarization curves of 1st, 500th, and 1000th sweep, and (**B**) CA curves CuS–NiTe_2_@PrGO in H_2_SO_4_ 0.5 M.

Stability is a crucial attribute for evaluating the performance of electrocatalysts used in the HER. To assess the stability of CuS–NiTe_2_@PrGO, we conducted 1000 consecutive sweeps of the HER process in a 0.5 M H_2_SO_4_ solution. Figure 9A illustrates the polarization curves obtained during the 1st, 500th, and 1000th sweeps over a potential range of 0–(–0.8) V (vs. RHE) at a scan rate of 30 mV s^–1^. As a result, the HER current density increased up to the 500th sweep due to factors such as enhanced surface porosity and the formation of active edge sites. Subsequently, between the 500th and 1000th sweeps, the current density remained constant.

To assess the catalytic stability of CuS–NiTe_2_@PrGO before and after 1000 sweeps, we employed the chronoamperometry (CA) technique, running it for 24 h at an applied potential of − 0.19 V (vs. RHE) in a 0.5 M H_2_SO_4_ solution (Fig. 9B). The results affirm that CuS–NiTe_2_@PrGO exhibits long–term durability in acidic environments. The LSV test was repeated three times, and the RSD of η_10_ for the 1st, 500th, and 1000th sweeps ranged from 3.4 to 5.2%. XRD analysis (Figure S2) displays that the structural integrity of the CuS–NiTe₂@PrGO catalyst was maintained even after 1000 catalytic sweeps, with no significant signs of sample degradation. However, a new peak was observed, likely attributed to surface reconstruction occurring during HER.

To avoid Pt-dissolution and deposition, a stability test (LSV) for 1000 sweeps were conducted using graphite as the counter electrode. As displayed in Figure S3, the plot trends are approximately same in both conditions. Moreover, after performing 1000 sweeps, the ICP-OES analysis indicates that the Pt amount is below 0.5 µg. Therefore, Pt as the counter electrode did not notably affect the HER results.

Finally, we compared the electrocatalytic performance of CuS–NiTe_2_@PrGO for HER in acidic conditions with previous electrocatalysts of similar metals (Table 2). Remarkably, this novel electrocatalyst exhibits outstanding HER performance without the inclusion of noble metals like Pd or Pt in its composition.

**Table 2:** Assessment of HER performance in the specified conditions, comparing our work with various related electrocatalysts.

Electrocatalyst	Tafel slope (mV dec^–1^)	η_10_ (mV)	Medium	References
Graphene/graphene nanoribbon aerogels	–183	49	H_2_SO_4_ 0.5 M	^43^
H–2D/3D–MoS_2_–rGO	–290	77	H_2_SO_4_ 0.5 M	^44^
N–rGO@Pt–Pd	–51	32	KOH 0.5 M	^45^
Core–shell Cu@Ni	–140	79	KOH 1.0 M	^46^
SrMoO_4_@ rGO/f–MWCNT	–139	81	H_2_SO_4_ 0.5 M	^47^
Fe–Ni/LDH@Au	–192	112	KOH 1.0 M	^48^
CoS_2_/MoS_2_@N–rGO–MWCNT	–281	73	H_2_SO_4_ 0.5 M	^49^
MoS–rGO	–280	77	H_2_SO_4_ 0.5 M	^50^
Ni–Co/LDH@NiCo_2_S_4_	–219	41	KOH 1 M	^51^
nP/Cu–Te	–250	36	KOH 0.5 M	^52^
CuS–NiTe_2_@PrGO	–106	57	H_2_SO_4_ 0.5 M	This work

## Conclusion

A novel nanocomposite, CuS–NiTe_2_@PrGO, was successfully synthesized and subjected to a comprehensive investigation of its electrocatalytic capabilities in promoting hydrogen production. The experimental findings unveiled the nanocomposite’s commendable electrocatalytic performance in the context of the HER under acidic conditions. Impressively, it exhibited a low overpotential of just − 106 mV at a current density of 10 mA cm^–2^, coupled with a favorable Tafel slope of 57 mV dec^–1^. What truly distinguishes the CuS–NiTe_2_@PrGO nanocomposite is its economic viability when compared to noble–metal–based electrodes. This cost–effectiveness, combined with the promising results it delivered and its notable durability, strongly advocates for the application of this nanocomposite in the domain of hydrogen evolution through acidic water electrolysis. The synthesis and characterization of CuS–NiTe_2_@PrGO offer a significant contribution to the field of electrocatalysis, providing a sustainable and financially feasible alternative for hydrogen production, which holds tremendous potential in advancing clean energy technologies.

## Methods

### Materials and reagents

Cupric nitrate (Cu(NO_3_)_2_·3H_2_O), nickel chloride (NiCl_2_·6H_2_O), thioacetamide (CH_3_CSNH_2_), phytic acid (C_6_H_18_O_24_P_6_), and potassium tellurium trioxide (K_2_TeO_3_) were purchased from Merck. potassium permanganate (KMnO_4_), sodium nitrite (NaNO_2_), sulfuric acid (H_2_SO_4_), hydrogen peroxide (H_2_O_2)_, and graphite powder were obtained commercially from Sigma–Aldrich.

### Characterization instruments

Field Emission Scanning Electron Microscopy (FE–SEM) images were obtained using a FEG 450 microscope (FEI QUANTA Co., USA) equipped for elemental mapping. Transmission electron microscopy (TEM) was performed on a CM120 instrument (PHILIPS Co., Netherlands). X–ray diffraction (XRD) patterns were collected using an AWXDM300 diffractometer (Asenware Co., China) to analyze crystal structure. Raman spectra were acquired on a Raman spectrometer (Teksan Co., Iran) to evaluate chemical bonding. X–ray photoelectron spectroscopy (XPS) was conducted on a VGESCALab220i–XL system (VG, USA) utilizing Al–Kα radiation to examine elemental composition. The ICP–OES was analyzed via Vista–PRO (Varian, USA).

### Electrochemical measurements

The measurements of HER were conducted using a Bio–Logic SP–300 Potentiostat/Galvanostat (France) at ambient temperature. A standard three–electrode setup was employed, with the auxiliary electrode being a Pt rod, the reference electrode being an Ag/AgCl electrode (with 3 M KCl solution), and the working electrode being a glassy carbon electrode (GCE). To clean the working electrode, a GCE was polished with alumina powder (0.05 μm) and then sonicated in a mixture of EtOH and H_2_O (1:1) for 5 min. To prepare a homogenized ink, 2.5 mg of the CuS–NiTe_2_@PrGO composite or other electrocatalysts were dispersed in 1 mL H_2_O and further sonicated for 30 min. Then, 10 µL of homogenized catalytic ink and 5 µL Nafion (2% in ethanol) were dropped on the cleaned surface of GCE and allowed to dry at room temperature, respectively.

To assess the performance of the HER, we recorded measurements using Linear Sweep Voltammetry (LSV), Chronoamperometry (CA), and Electrochemical Impedance Spectroscopy (EIS) techniques in a 0.5 M H_2_SO_4_ solution within the relevant potential range, relative to the reversible hydrogen electrode (RHE), in accordance with the Nernst equation as follows,

$$E\left( {V\;vs.{\text{ }}RHE} \right) = E\left( {V\;vs.\;{\text{Ag}}/{\text{AgCl}}} \right) + 0.059\;{\text{pH}} + E^{0} _{{{\text{Ag/AgCl}}}}$$

The Tafel equation constructed the Tafel plots;

$$\eta = b\; log \;j + a$$

where *j* is the current density, *b* is the Tafel slope, and a is the intercept relative to the exchange current density.

### Preparation of 3D PrGO

GO was synthesized by the Hummers’ method as described previously^53^. To prepare the 3D PrGO, 120 mg of the as–prepared GO was dispersed in 60 mL of distilled water using probe sonication for 1 h. 100 mg of phytic acid (C_6_H_18_O_24_P_6_) (2.52 mM) was then added to the GO dispersion as a phosphorus dopant source. The mixture was ultrasonicated for another hour to ensure uniform dispersion. The resultant mixture was transferred to a 100 mL autoclave and hydrothermally treated at 170 °C for 12 h. Finally, the obtained 3D PrGO product was washed 5 times with a 1:1 v/v ethanol/water solution and freeze–dried for 24 h.

### Preparation of CuS–NiTe2@PrGO

Firstly, 40 mg of PrGO was added to 60 mL of distilled water and sonicated well for 2 h. After the solution was homogenized, 24 mg of (NiCl_2_·6H_2_O) and 24 mg of (Cu(NO_3_)_2_·3H_2_O) were added to it. Then under vigorous stirring, 12 mg (K_2_TeO_3_) and 4 mg (CH_3_CSNH_2_) were added to the solution. After stirring the mixture for 30 min, it was steadily heated to 90 °C at a rate of 2 °C/min and maintained at this temperature for 3 h. Upon cooling to room temperature, the precipitate was washed with distilled water until it reached neutral pH. The washed precipitate was vacuum–dried at 60 °C for 24 h and labeled as CuS–NiTe_2_@PrGO. For the synthesis of CuS–NiTe_2_@PrGO used GO instead of PrGO. For the synthesis of NiTeS and CuTeS, the same method was used, only Cu(NO_3_)_2_·3H_2_O for NiTeS and NiCl_2_·6H_2_O for CuTeS were removed, respectively.

## Electronic supplementary material

Below is the link to the electronic supplementary material.

Supplementary Material 1

## Data Availability

The datasets supporting the conclusions of this article are included within the article.
