# Supplementary material for: CuS-NiTe2 embedded phosphorus-doped graphene oxide catalyst for evaluating hydrogen evolution reaction
Source: Sci Rep. 2024 Nov 11;14:27622. doi: 10.1038/s41598-024-78870-w (PMC11554888; doi:10.1038/s41598-024-78870-w)
Supplement: Supplementary file 1 — Supplementary Material 1 [file 41598_2024_78870_MOESM1_ESM.docx]

**CuS-NiTe_2_ Embedded Phosphorus-doped Graphene Oxide Catalyst for Evaluating Hydrogen Evolution Reaction**

**Sedigheh Parvaz^1^, Zahra Talebi Vandishi^1^, Ali A. Ensafi^[[1]](#footnote-1),2^, Kimia Zarean Mousaabadi*^,1^**

*1. Department of Chemistry, Isfahan University of Technology, Isfahan 84156-83111, IRAN*

*2. Adjunct Professor, Department of Chemistry & Biochemistry, University of Arkansas, Fayetteville, AR 72701, USA.*

**Figure. S1** Nyquist plots a CuS–NiTe_2_@PrGO (R_ct2_ = 18 Ω), CuS–NiTe_2_@rGO (R_ct2_ = 33 Ω), CuS–NiTe_2_ (R_ct2_ = 45 Ω), NiTeS (R_ct_ = 69 Ω), and CuTeS (R_ct_ = 107 Ω) at −135 mV.

**Figure. S2** XRD pattern of CuS–NiTe_2_@PrGO after 1000 sweeps.

**Figure. S3** Polarization curves of 1000^th^ sweep of CuS–NiTe_2_@PrGO with graphite and platin as a counter electrode in H_2_SO_4_ 0.5 M.


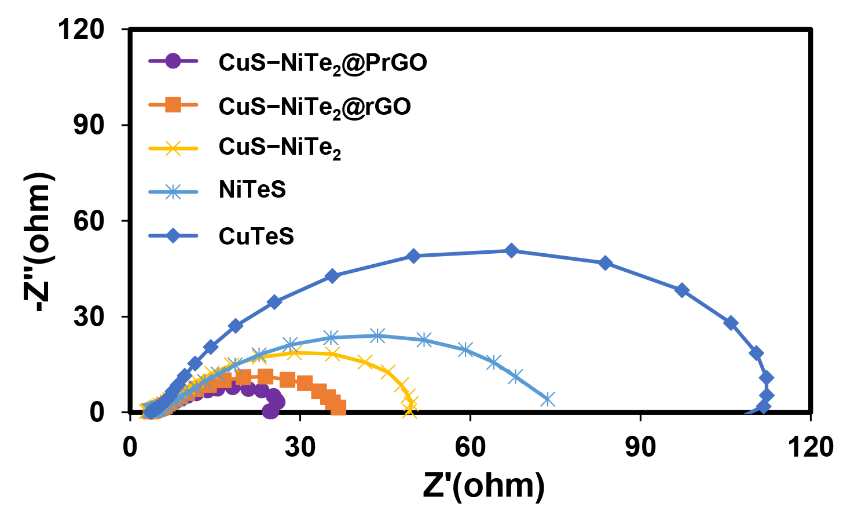


**Figure. S1** Nyquist plots a CuS–NiTe_2_@PrGO (R_ct2_ = 18 Ω), CuS–NiTe_2_@rGO (R_ct2_ = 33 Ω), CuS–NiTe_2_ (R_ct2_ = 45 Ω), NiTeS (R_ct_ = 69 Ω), and CuTeS (R_ct_ = 107 Ω) at −135 mV.


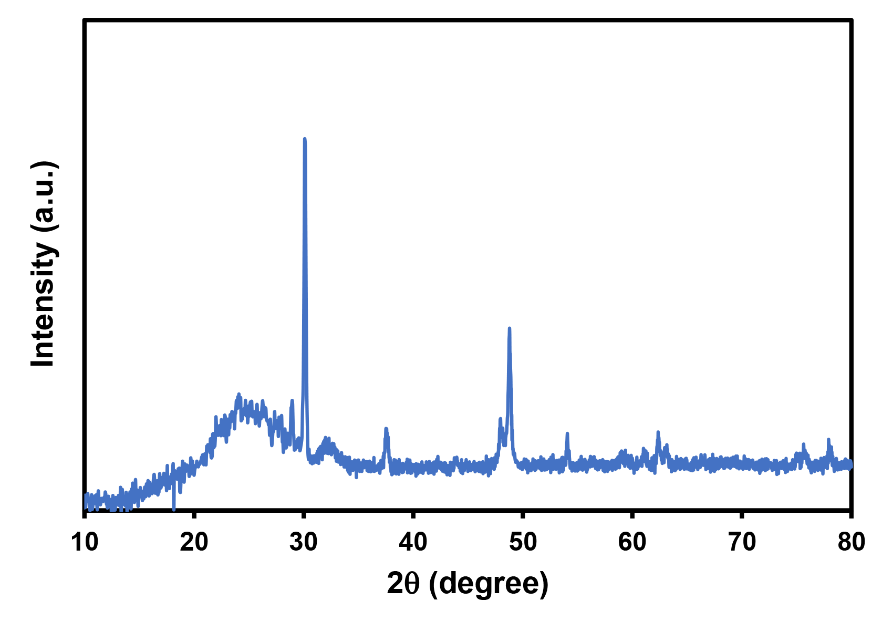


**Figure. S2** XRD pattern of CuS–NiTe_2_@PrGO after 1000 sweeps.


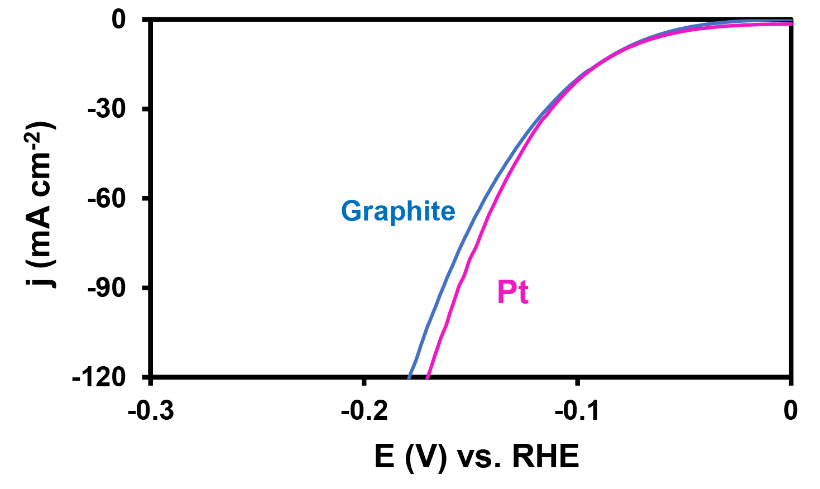


**Figure. S3** Polarization curves of 1000^th^ sweep of CuS–NiTe_2_@PrGO with graphite and platin as a counter electrode in H_2_SO_4_ 0.5 M.

1. * Corresponding author: Tel.; +45-91470477; E-mail: kimia.zarean@gmail.com. [↑](#footnote-ref-1)
